# Neuroprotective Effect of Melatonin against Kainic Acid-Induced Oxidative Injury in Hippocampal Slice Culture of Rats

**DOI:** 10.3390/ijms15045940

**Published:** 2014-04-09

**Authors:** Hyung A Kim, Kyung Hee Lee, Bae Hwan Lee

**Affiliations:** 1 Department of Physiology, Brain Korea 21 Project for Medical Science, Brain Research Institute, Yonsei University College of Medicine, Seoul 120-752, Korea; E-Mail: electrolab@daum.net; 2 Division of Health Science, Department of Dental Hygiene, Dongseo University, Busan 617-716, Korea; E-Mail: kyhee@gdsu.dongseo.ac.kr

**Keywords:** melatonin, kainic acid, organotypic hippocampal slice culture, reactive oxygen species, antioxidant, neuroprotection

## Abstract

Endogenous melatonin is a known free radical scavenger that removes reactive oxygen species (ROS), thus, alleviating oxidative stress. The purpose of this study was to demonstrate its effect against kainic acid (KA)-induced oxidative stress in organotypic hippocampal slice cultures (OHSCs). To observe neuroprotective effects of melatonin, different concentrations (0.01, 0.1 and 1 mM) of melatonin were administrated after KA treatment for 18 h in OHSCs of rat pups. Dose-response studies showed that neuronal cell death was significantly reduced after 0.1 and 1 mM melatonin treatments based on propidium iodide (PI) uptake and cresyl violet staining. The dichlorofluorescein (DCF) fluorescence which indicates ROS formation decreased more in the melatonin-treated group than in the KA group. The expression of 5-lipoxigenase (5-LO) and caspase-3 were reduced in the melatonin-treated groups compared to the KA group. These results suggest that melatonin may be an effective agent against KA-induced oxidative stress in the OHSC model.

## 1. Introduction

It is well known that the excessive production of oxygen free radicals may generate neuronal disorders. The brain is one of the most oxygen consuming organs [1]; therefore, reactive oxygen species (ROS) production in the brain is higher than in the rest of the body. In general, an antioxidant maintains and regulates the equilibrium between ROS formation and extinction. If the equilibrium is disrupted, however, ROS act as a stressor and damage intracellular elements such as proteins, lipids, and DNA. Because neuronal cells are especially sensitive and vulnerable to ROS, apoptosis or necrosis can occur easily [2]. Therefore, oxidative stress produces neurotoxicity in the brain. Specially, mitochondrial oxidative stress has frequently been associated with various neuronal disorders [3].

Melatonin is a known antioxidant secreted from various organs, including the pineal gland, retina, lens, and gastrointestinal tract. Melatonin plays a variety of physiological roles such as the adaptation of the day and night cycle, acclimation, and participation in immune reaction. Its protective effect in many neurodegenerative disorders (e.g., Alzheimer’s disease, Parkinson’s disease, ischemia-reperfusion injury, mental disorders) have also been implicated [4–7]. Because of its favorable physicochemical proprieties, melatonin is able to pass biological barriers such as the cellular membrane [8]. In addition, because it is an electron-rich molecule, melatonin produces stabilized molecules by reacting with various ROS. Particularly, melatonin produces stabilized molecules without generating any other ROS because redox cycling is not required for melatonin. Melatonin is referred to as a suicidal or terminal antioxidant, because the body excretes metabolites produced by melatonin, via urine, without any secondary influence on body tissue [9]. Melatonin binds to the membrane receptor with high affinity in the picomolar range, and also to the nuclear receptor as well as calmodulin in the nanomolar range [10]. Melatonin has a free radical scavenging function at even higher concentrations. As a result, melatonin acts as an antioxidant [8,11–14].

Kainic acid (KA) is an agonist of the ionotrophic glutamate receptor. Its action as an excitotoxin leads to neuronal excitotoxicity and oxidative damage in the central nervous system [15]. KA binds to the kainate receptor and produces neuronal death [16]. Therefore, KA has been used to study the neuroprotective effects of various agents against oxidative stress.

In our previous studies, we have shown that antioxidants, such as Coenzyme Q10 and ascorbic acid, have elicited neuroprotective effects against KA-induced neurotoxicity in organotypic hippocampal slice culture (OHSC) [17–19]. The goal of the present study was to investigate the ability of melatonin to protect cell viability in the same *in vitro* model system.

## 2. Results and Discussion

### 2.1. Effects of Melatonin on KA-Induced Neuronal Toxicity in OHSCs

**Figure 1.**
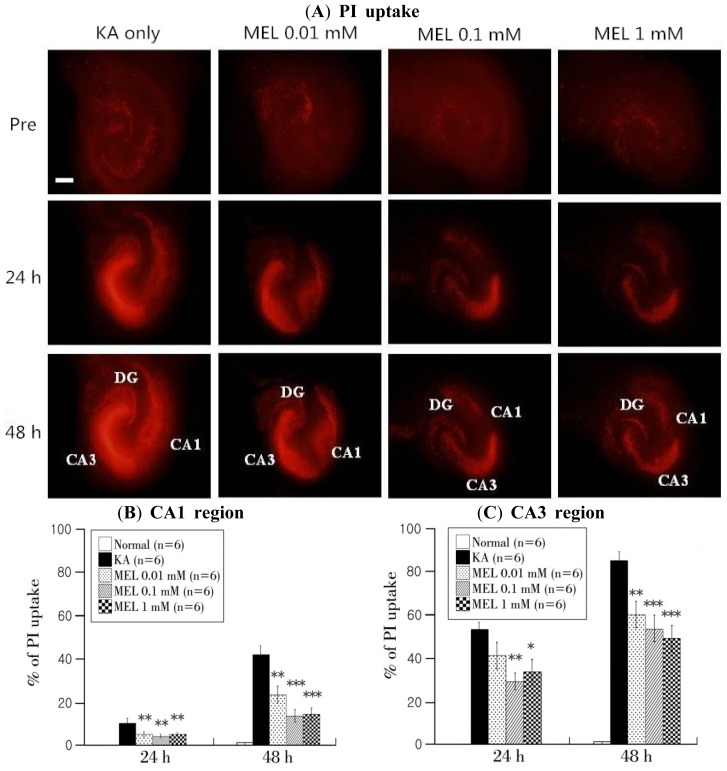
Melatonin reduces KA-induced neuronal injury in organotypic hippocampal slice cultures (OHSCs). (**A**) Difference of PI uptake fluorescence intensities in KA only-treated group and melatonin-treated groups determined using a fluorescence microscope; (**B**,**C**) Quantification of PI uptake intensities using a MetaMorph program in the CA1 (**B**) and CA3 (**C**) regions. The horizontal axis indicates time after melatonin treatment and the vertical axis represents the percent of PI uptake in each region. Fluorescence values were calculated using the following formula: % of PI uptake = 100 × (*F*_t_ − *F*_pre_)/(*F*_fk_ − *F*_pre_). Data are mean ± SEM (*n* = 6). * *p* < 0.05, ** *p* < 0.01, *** *p* < 0.001: one-way ANOVA followed by a LSD test for the comparison with the KA only-treated group. Scale bar: 500 μm.

PI uptake was used to investigate the effects of melatonin on KA-induced neuronal injury. Hippocampal tissues showed almost no damage in the pre-phase. The intensity of PI uptake was increased in the hippocampus CA1, CA3, and dentate gyrus 18 h after KA treatment. However, reduced intensity was shown in 0.01, 0.1, and 1 mM melatonin-treated groups compared with the KA only-treated group (Figure 1A). The values in % of PI uptake in the CA1 and CA3 region of the hippocampus layers are shown in Figure 1B,C. Reduced levels of PI uptake in CA1 and CA3 were observed in the 0.01, 0.1, and 1 mM melatonin-treated groups.

### 2.2. Cresyl Violet Staining

**Figure 2.**
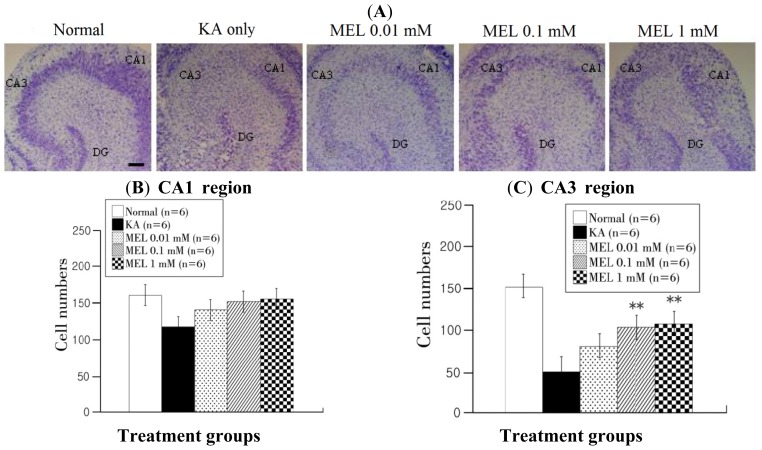
Melatonin increases neuronal survival in KA-induced neuronal injury in OHSCs. (**A**) Morphology of survived cells in 10 μm dissected hippocampal tissue. The pictures represent normal, KA only treated, 0.01, 0.1, and 1 mM melatonin-treated groups at 48 h after melatonin treatment; (**B**,**C**) Quantification of cell survival using a MetaMorph program in CA1 (**B**) and CA3 (**C**) regions. The horizontal axis indicates treatment groups and the vertical axis indicates the number of cresyl violet-positive cells. Data are mean ± SEM (*n* = 6). ** *p* < 0.01: one-way ANOVA followed by LSD test for the comparison with KA only-treated group. Scale bar: 200 μm.

Cresyl violet staining was used to detect neuronal cell survival. Normal tissue had numerous neuronal cells throughout all the cell layers. KA only-treated tissue had fewer cells in the layer, especially in the CA3 region compared with the CA1 region. More cells survived in melatonin-treated than in KA only-treated tissue, but this number was lower than that in normal tissue (Figure 2).

### 2.3. Formation of ROS in KA-Induced Oxidative Stress

**Figure 3.**
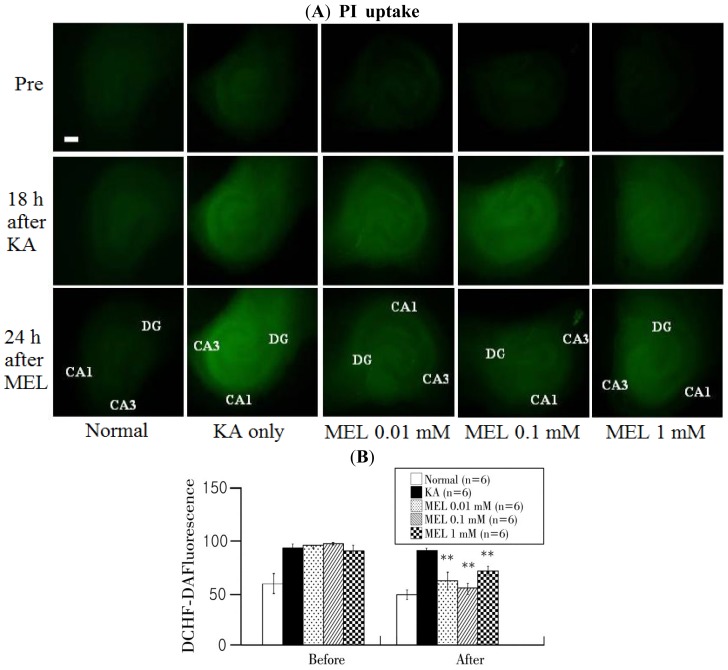
Relationship between melatonin treatment and ROS formation. (**A**) DCFH-DA intensities in each group. DCFH-DA expression level was detected at pre, 18 h after KA injury, and 24 h after melatonin treated phase; (**B**) Quantification of DCFH-DA intensities using a MetaMorph program. The horizontal axis indicates pre-melatonin treatment (Before) and 24 h after melatonin treatment (After) and the vertical axis represents DCFH-DA intensity in the hippocampal tissues. Data are mean ± SEM (*n* = 6). ** *p* < 0.01: one-way ANOVA followed by LSD for comparison with each group. Scale bar: 500 μm.

DCFH-DA fluorescence dye was used to detect ROS formation in the hippocampal tissue (Figure 3). ROS formation was detected from the overall cell layers. 10 μM dye was represented in a green fluorescence. Fluorescence in the tissue was minute before the KA treatment, but 5 μM KA generated fluorescence beyond normal. After melatonin treatment, the fluorescence was significantly reduced compared with KA-treated groups (*p* < 0.01).

### 2.4. Western Blotting

**Figure 4.**
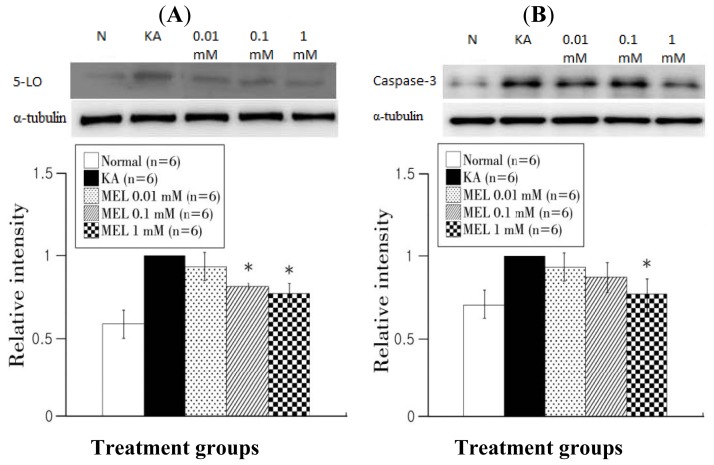
Western blot analysis of 5-LO and caspase-3 in OHSC after melatonin treatment following KA. (**A**) The expression of 5-LO at 24 h after the 0.1 and 1 mM melatonin-treated groups was significantly lower than that of the KA-treated group; (**B**) The level of caspase-3 at 24 h after melatonin treatment. The 1 mM melatonin-treated group showed lower caspase-3 expression than KA group. The horizontal axis indicates each experimental group (Normal, KA, 0.01, 0.1, 1 mM) and the vertical axis represents the level of protein expression ratio (antibody expression/α-tubulin expression). Data are mean ± SEM. * *p* < 0.05: one-way ANOVA followed by an LSD test.

The 5-lipoxigenase (5-LO) expression is a marker of lipid peroxidation. The KA group had higher 5-LO expression than the other groups (Figure 4A). Twenty-four hours after melatonin treatment, 0.1 and 1 mM of melatonin significantly reduced the 5-LO expression. Caspase-3 expression is a marker of apoptosis, and also plays a role in necrosis and inflammation. The normal group showed lower expression than the KA-treated group. The 0.01, 0.1, and 1 mM melatonin-treated groups also showed reduced caspase-3 expression (Figure 4B).

### 2.5. Discussion

It has been previously shown that KA generates ROS in the brain [20,21]. Recently, some studies have demonstrated that melatonin may attenuate brain injury through its antioxidant effect. According to Juliana *et al.* [22], amyloid-β neurotoxicity was reduced by melatonin in organotypic culture. It has also been shown that the administration of melatonin in rats attenuates oxidative stress-induced neurodegeneration and microglial generation [23]. Especially, Zaja-Milatovic *et al.* [20] have noticed that MT1A and MT1B receptors are abundant in the CA1 and CA3 region of the hippocampus which regulates the activation of melatonin.

In our study, to demonstrate the antioxidant effects of melatonin, the KA-induced oxidative stress model was chosen because the KA-induced injury is more effective in the hippocampal CA3 region than CA1 which makes it easy to specify an injury model [24–26]. There was no PI uptake observed at different concentrations (0.01–1 mM) of melatonin alone in the OHSC (data not shown). This study would be a valuable support for previous studies related to antioxidant effects of melatonin on KA-induced oxidative stress. The results demonstrated that melatonin effectively suppresses neuronal cell death in CA1 and CA3 regions in the hippocampus, based on measurement of PI uptake.

The DCFH-DA method is widely used in ROS measurement in the mitochondria. It is a major advantage of DCFH-DA that it detects both mitochondria and live cells [27]. The results from fluorescence measurements using DCFH-DA demonstrated the association between neuronal death and ROS. We also found a relationship between PI and cresyl violet staining. Unlike the ROS generation, the results from PI uptake and cresyl violet staining were dependent on melatonin concentration, which increased as the concentration of melatonin increased.

According to Flavia *et al.* [28], KA-induced damage showed up-regulation of 5-LO expression in limbic system. In addition, increased endogenous ROS up-regulated 5-LO expression. The generation of the endogenous ROS by antimycin A increases up-regulation of 5-LO activity three-fold [29]. Apoptotic neuronal death with KA injury induces rapid induction of bax, collapse of mitochondrial membrane potential, release of cytochrome c from mitochondria, and activation of caspase-3, suggesting that multiple apoptotic pathways are activated in response to KA [30]. Caspase-3 participates in various apoptosis pathways including KA-induced injury. From our study, melatonin gradually reduced caspase-3 activity, especially, that 1 mM melatonin significantly attenuated in caspase-3 expression. This finding indicates that melatonin has neuroprotective effects that regulate viability of cells against ROS and apoptosis. Several studies have demonstrated that antioxidants can attenuate KA-induced neuronal damage [31,32]. Especially, mitochondria superoxide radical production generates ROS and hippocampus destruction demonstrated that the oxygen free radical was generated in the limbic structure by KA-induced seizures, and another study demonstrated that KA-induced damage in neuron generated a mitochondrial superoxide production [20,23,33].

Taken together, this study demonstrated the effect of melatonin on KA-induced oxidative damage using different concentrations of melatonin. Treatment with melatonin significantly attenuated neuronal damage and the generation of ROS. Higher concentrations of melatonin were more effective than that of lower concentrations. These results suggest that melatonin could be an effective antioxidant in the context of oxidative stress.

## 3. Experimental Section

### 3.1. Organotypic Hippocampal Slice Culture (OHSC)

All animal experiments were approved by the Institutional Animal Care and Use Committee of Yonsei University Health System. OHSC was conducted in accordance with our previous experimental protocols [16–18] modified from Stoppini *et al*. [34]. In brief, the brains of 6–8 day-old Sprague-Dawley rats (Koatech, Gyeonggi-do, Korea) were removed and transferred to Gey’s Balanced Salt Solution (GBSS, Sigma, St. Louis, MO, USA) containing 0.5% glucose and 3 mM KCl, 1 N HCl. Rat hippocampi were separated from the whole brains and then dissected to 350 μm with a McIlwain tissue chopper (Vibratome, O’Fallon, MO, USA). Undamaged and clear layered dissected tissues were selected. Six to eight tissues were transferred onto a Millicell-CM membrane insert (Millipore, Billerica, MA, USA) in 6-well plate containing 1 mL culture media (pH 7.2) composed of 50% Opti-MEM, 25% Hank’s Balanced salt solution (HBSS), 25% heat inactivated horse serum (all from GIBCO BRL, Grand Island, NY, USA), and 6.5 mg/mL d-glucose (AMRESCO Inc., Solon, OH, USA). Cultured slices were incubated at 36 °C in a humidified atmosphere of 5% CO_2_. Culture media was changed three times a week. Slices were grown for 3 weeks in the culture medium.

### 3.2. Drug Preparation and Treatment

**Figure 5.**
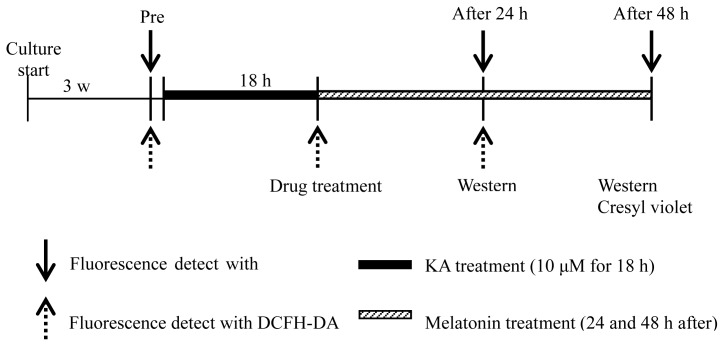
Diagram of the experimental procedure. OHSCs was cultured for three weeks and 5 μg/mL PI was treated in the culture medium (pre-phase, Pre). After 1 h, the pictures of pre-phase cultured hippocampus were taken. Then, 10 μM DCFH-DA was treated for 30 min and DCFH-DA picture was taken. KA was diluted and treated with fresh culture medium for 18 h. DCFH-DA pictures were taken in this phase but not PI uptake. Subsequently, melatonin-treated hippocampus pictures of 24 and 48 h after melatonin treatment for PI and 24 h DCFH-DA were gathered.

Five micro moles per liter kainic acid (KA, K0250, Sigma, St. Louis, MO, USA) was dissolved in DW and applied to cultured slices for 18 h in OHSC. Melatonin (Sigma) was dissolved in ethanol (Merk, Darmstadt, Germany) then diluted and treated with culture medium at 0.01, 0.1, and 1 mM concentrations. Afterwards, KA-injured hippocampal slices were kept on melatonin for 24 and 48 h. The experimental procedure is shown in Figure 5.

### 3.3. Measurement of Neuronal Injury

To observe neuronal cell death, we used propidium iodide (PI, Sigma, St. Louis, MO, USA) staining. Three weeks after OHSCs, cultured normal hippocampal slices were added with 5 μM PI in culture medium and detected the neuronal death in the pre-phase which has no PI uptake in the hippocampal layers. After KA treatment for 18 h, degenerated tissue was removed from the membrane insert and PI uptake was monitored. Effects of 0.01, 0.1, and 1 mM melatonin treatment for 24 and 48 h were observed by PI. Full kill was performed with 10 mM NMDA (Sigma) to produce neuronal death in all tissue areas. Finally, pre, 24 h, 48 h, and full-kill phase results were detected by the fluorescence microscope and analyzed with a MetaMorph program (Universal Imaging, Downingtown, PA, USA). All values of the fluorescence were calculated using the following formula: % of PI uptake = (*F*_t_ − *F*_pre_)/ (*F*_fk_ − *F*_pre_) × 100 (*F*_t_ = PI uptake at 24 or 48 h after melatonin treatment, *F*_pre_ = pre, *F*_fk_ = full kill).

### 3.4. Evaluation of Intracellular ROS Formation

Tissues were incubated with 10 μM 2′,7′-dichlorohydrofluorescein diacetate (DCFH-DA, Sigma) in a 36 °C humidified incubator for 30 min and washed twice with phosphate-buffered saline, pH 7.4 (1× dPBS, GIBCO, Grand Island, NY, USA). DCFH-DA expression level was detected at pre, 18 h after KA injury and 24 h after melatonin treatment using a fluorescence microscope (IX-71, Olympus, Tokyo, Japan). The DCFH-DA fluorescence intensity was measured by a MetaMorph program (Universal Imaging).

### 3.5. Cresyl Violet Staining

To detect the survival of cells using the cresyl violet staining, each slide was soaked in 4% paraformaldehyde (Duksan, Incheon, Korea) for 20 min, washed out twice with 1× dPBS, soaked into cresyl violet solution (Sigma), and washed with flowing tap water. The tissue sections were dehydrated with 70%, 90%, and 100% EtOH (Ducksan), soaked into the 50:50 (EtOH and xylene) mixture, and stored in 100% xylene (Ducksan). Finally the slides were mounted with permount. The MetaMorph program was used to detect the survived neurons in CA3.

### 3.6. Western Blot Analysis

Cultured slices were collected and homogenized with lysis buffer containing 10% SDS, 1 M Tris–HCl (pH 7.4), 5% Triton X-100, 50% sodium deoxycholate, 1 M DTT, 0.5 M sodium orthovanadate, 2 mg/mL PMSF, 10× protease inhibitor (all from Sigma). The homogenized solution was frozen and thawed in −80 °C three times. Proteins were separated by SDS-PAGE, and transferred to a nitrocellulose membrane (Millipore, Billerica, MA, USA). The membrane was blocked by 5% skim milk in Tris-buffered saline added with 0.5% Tween-20, and was incubated with primary antibodies (5-LO, Cayman, Ann Arbor, MI, USA; caspase-3, Santacruz Biotechnology, Santa Cruz, CA, USA; β-actin, Abcam, Cambridge, UK) overnight at 4 °C. The membrane was developed with a peroxidase-conjugated secondary anti body, and protein was detected by enhanced chemiluminescence (ECL) procedure (Amersham, Arlington, IL, USA).

### 3.7. Statistical Analysis

All data were expressed as mean ± SEM. Differences among groups were analyzed by LSD *post-hoc* test. The level of significance was accepted at * *p* < 0.05, ** *p* < 0.01 and *** *p* < 0.001.

## 4. Conclusions

The present study used PI, cresyl violet, and DCFH-DA fluorescence staining to demonstrate that KA-induced injury is related to generation of ROS, which has a neurotoxic effect in the hippocampus. We found that melatonin reduced ROS generation and neuronal cell death. This suggests that melatonin has neuroprotective effects on KA-induced oxidative stress in the hippocampus as an antioxidant. Melatonin may therefore be important for alleviating neuronal disorders that can be generated by oxidative stress.
